# Risk factors for rectal bleeding in prostate cancer after radiotherapy with a validation of current rectal dose constraints

**DOI:** 10.2340/1651-226X.2025.42551

**Published:** 2025-05-08

**Authors:** Ellen Lund Schaldemose, Christine Vestergaard Madsen, Ahmed Hussein Zedan, Martin Berg, Henrik Dahl Nissen, Terje Andersen, Bjarke Mortensen, Lars Ulrik Fokdal

**Affiliations:** a Department of Oncology, Vejle Hospital, University Hospital of Southern Denmark, Vejle, Denmark; b Radiotherapy Research Team, Department of Oncology, Vejle Hospital, University Hospital of Southern Denmark, Vejle, Denmark

**Keywords:** Rectal bleeding, curative radiotherapy, prostate cancer

## Abstract

**Background:**

Rectal bleeding is a well-known adverse event following pelvic external beam radiotherapy (EBRT) for prostate cancer. This study investigates risk factors for rectal bleeding and validate our current rectal dose constraints in a real-world setting.

**Material and methods:**

This prospective study includes 248 prostate cancer patients who received EBRT between 2017-2022. EBRT consisted of 56 Gy/39 fractions to the prostate, elective lymph nodes, and seminal vesicles with an integrated boost of 78 Gy to the prostate alone (≤T3a) or to the prostate and seminal vesicles (T3b). Rectal dose constraints were V50 Gy ≤50%, V70 Gy ≤20%, and V74 Gy ≤12%. Rectal bleeding was recorded at baseline and regularly duringfollow-up and included staff reported morbidity and patient reported outcome measures. Risk factors were evaluated in multivariate cox regression analysis.

**Results:**

Median follow-up was 18 months (range 1-61 months). Sixteen percent (CI:11%;22%) of patients reported rectal bleeding as “rarely”, 4%(CI:2%;8%) “about half the time”, 0% “usually”, and 2%(CI:0%;4%) “always”. Five percent reported rectal bleeding as bothersome.

It was possible to comply with current rectal dose constraint (V74 Gy ≤12%) in 99.6% of all patients. Body mass index (BMI) (BMI:25-29.9, HR:0.54(CI:0.30;0.98), p=.044 or BMI>29.9, HR:0.40(CI:0.20;0.79), p=0.008)) were predictors for rectal bleeding.

**Interpretation:**

Patient-reported rectal bleeding is common after prostate cancer radiotherapy. High BMI was a protective factor against rectal bleeding. No correlation was observed between rectal dose-volume constraints and the occurrence of rectal bleeding, suggesting that a rectal high-dose constraint of V74 Gy ≤12% is an adequate threshold to minimize patient-reported rectal bleeding.

## Introduction

Rectal bleeding is a well-known adverse event after external beam radiotherapy (EBRT) for prostate cancer with a cumulative incidence of 4%–12% depending on EBRT treatment, follow-up time, and study design [1–4].

Previous studies demonstrated that radiotherapy dose-volume parameters are associated with rectal bleeding [5, 6], but why some individuals develop rectal bleeding and others do not is not fully elucidated. Thus, conflicting results regarding potential risk factors such as cardiovascular disease, androgen deprivation therapy (ADT), diabetes, age, and use of anticoagulants/antiaggregants have been published [3, 7–10].

Even though rectal bleeding may be temporary and self-limiting [11], an improved understanding of risk factors for rectal bleeding is necessary to guide for decision-making processes and help in the development of preventive strategies.

In the last two decades, several large studies have investigated the relationship between radiotherapy dose and rectal bleeding following pelvic irradiation [6, 12–15]. The studies have concluded that the risk for the development of late rectal bleeding grade ≥2 (i.e. intermittent bleeding) according to the toxicity criteria from the radiation therapy oncology group (RTOG) is similar to an a priori risk of rectal bleeding if the rectal volume irradiated with 75 Gy (*V*_75 Gy_) is less than 12% [3, 12, 14, 15]. These results are contrary to a study by Fiorino et al. [16], where a more strict rectal dose constraint was found with a *V*_75 Gy_ ≤ 5% . However, this study investigates a much smaller patient population with shorter follow-up compared to other studies [12]. In light of the results outlined earlier, we have established a constraint for prostate cancer radiotherapy to ensure that the rectal volume receiving a dose of 74 Gy or higher remains below 12% (*V*_74 Gy_ ≤ 12%).

This prospective study investigates the incidence and risk factors for rectal bleeding in patients with prostate cancer receiving curative EBRT. Moreover, the impact of rectal bleeding on quality of life is investigated. Finally, our current rectal dose constraints for prostate cancer EBRT are validated in a real-world clinical setting within a consequetive cohort of patients.

## Methods and materials

### Study design and patients

In the Department of Oncology, Vejle Hospital, Denmark, 421 patients with prostate cancer treated with curative EBRT have been included in a prospective, observational study in the period from 2017 to 2022. Inclusion criteria for the present study were EBRT for primary prostate cancer and at least one registration of patient-reported outcome on rectal bleeding during the observation period (up to 60 months). A total of 248 patients fulfilled these inclusion criteria and were included. Diagnosis, staging, and risk group classification were determined according to the European Association of Urology guidelines [17, 18]. This study was reported to the Data Protection Agency (2008-28-0035) and conducted according to the declaration of Helsinki II. A written-informed consent was obtained from all patients.

### Treatment

**Figure 1:**
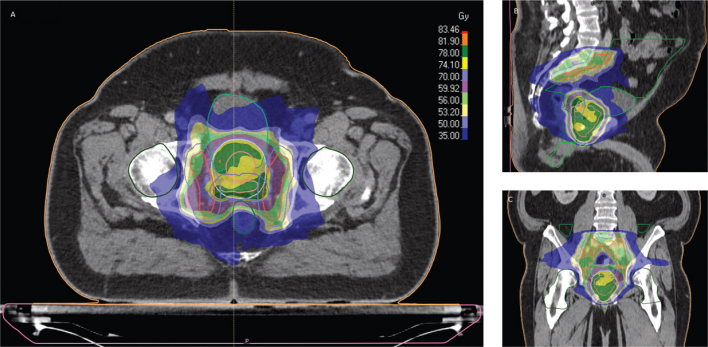
Dose distribution for a patient receiving 56 Gy to the elective lymph nodes and 78 Gy to prostate and seminal vesicles in transversal (A), sagittal (B), and coronal section (C). Clinical and planning target volumes are shown for elective lymph nodes, prostate, and seminal vesicles. Organs at risk include femoral heads, bladder, bowel cavity, penile bulb, testicles (not shown), anal canal and rectum.

All patients were treated with volumetric arc therapy (VMAT) using magnetic resonans imaging (MRI) as a single-modality solution (MR for Calculating Attenuation (MRCAT)). Patients with intermediate- or high-risk disease were treated with 6 or 36 months of ADT. The EBRT schedule consisted of 56 Gy/39 fractions to the prostate, elective lymph nodes, and seminal vesicles with a simultaneous integrated boost to a total dose of 78 Gy to the prostate alone in patients with ≤T3a disease or to the prostate and seminal vesicles in patients with T3b disease (Figure 1).

Before MRI for radiotherapy contouring and dose planning, the patient underwent bowel preparations with a fast-acting enema to alleviate rectal constipation. The rectum was delineated from the pelvic floor/anal canal to the rectosigmoid junction, incorporating both the muscular wall and the contents of the organ (definition R8 from Nitshce et al.) [19] Contouring of targets and organ at risk (OAR) were only done by consultants in clinical oncology who had successfully completed a ‘dummy run’, which involved contouring 10 cases to guarantee optimal consistency.

Dose constraints for the rectum were *V*_50 Gy_ ≤ 50%, *V*_70 Gy_ ≤ 20%, and *V*_74 Gy_ ≤ 12% based on the recommendations from Tucker et al. [14]. Daily positioning was based on gold markers in the prostate. For prostate and seminal vesicles, the planning target volume (PTV) margins were 7 mm in the transversal plane and 10 mm in the craniocaudal plane, while the PTV margins for the elective lymph nodes were 7 mm in the anteroposterior direction, 8 mm in the craniocaudal direction, and 5 mm laterally.

Before each radiotherapy fraction, a cone-beam-computed tomography scan (CBCT) was done to verify the position of the prostate and organs at risk. If notable changes in rectal volume or position were detected on the CBCT, patients were instructed to use an enema or laxatives to minimize these discrepancies.

### Endpoints and follow-up period

Patient-, disease-, and treatment-related data were registered at baseline (BL). Patient- and staff-reported morbidity were recorded at BL, at the end of treatment (EOT), and at regular intervals during a 5-year follow-up (4 weeks, 3 months(m), 6 m, 9 m, 12 m, 18 m, 24 m, 30 m, 36 m, 48 m, and 60 m).

Patient-reported outcome measures (PROMs) were assessed using the Expanded Prostate Cancer Index Composite (EPIC) [20]. Patient-reported rectal bleeding was measured using EPIC item 11: ‘How often have you had bloody stools during the last 4 weeks’, and the patients were instructed to choose between five ratings: ‘never’, ‘rarely’, ‘about half of the time’, ‘usually’, or ‘always’. To assess clinically significant bleeding bother, we used EPIC item 15e ‘How big a problem, if any, has each of the following been for you (e; Bloody stools)?’ with the following ratings: ‘no problem’, ‘very small’, ‘small’, ‘moderate’, or ‘big’ problem. In both questions, patients were asked to consider the past 4 weeks.

Systematic collection of staff-assessed bowel morbidity was done according to Common Terminology Criteria for Adverse Events (CTCAEs) v4.0, and the patients were asked to recall any events since their last visit in the outpatient clinic [21].

### Other endpoints

The use of antithrombotic medication and anticoagulants prescriptions was retrospectively collected using patient registries. Furthermore, all data on endoscopy examinations (indication, diagnose, and main findings) during follow-up were registered.

### Statistics

Stata for Windows (version 11.2; IMB Corp., Armonk, NY) was used for data analysis. Continuous variables are presented as median with interquartile range (IQR) and categorical variables as numbers and proportions. Prevalence of rectal bleeding at BL and each follow-up was calculated. The Wilcoxon signed-rank test was used to evaluate the difference in rectal bleeding between BL and follow-up.

Nelson-Aalen cumulative hazard curves were constructed to estimate time to event distributions from the EOT (i.e. BL morbidity was not included). Patients were censored at the date of the last follow-up or at disease recurrence or death. Trends in rectal bleeding (defined as at least ‘rarely’) were explored using univariate and multivariate Cox regression analyses. The following BL patient characteristic were included as possible risk factors: age, World Health Organization (WHO) performance status (PS), Charlson Comorbidity Index (CCI) (19 conditions and associated weights, combined with age), BMI, diabetes, tobacco, and anticoagulant or antiaggregant medication. Furthermore, the following BL disease and dosimetric characteristics were included: prostate-specific antigen (PSA), Gleason score, T-stage, ADT, prostate volume, high-dose radiotherapy targets (prostate alone, prostate + seminal vesicles), rectum *V*_70 Gy_, and rectum *V*_74 Gy_. Finally, selected CTCAE (any grade due to very few reports of grade > 1) assessed morbidity at every follow-up: hemorrhoids (no, yes) and obstipation (no, yes). All factors with *p* < 0.1 were included in the multivariate analysis.

The Pearson’s chi^2^ test was used to compare rectal bleeding frequency (EPIC item 11) with the bleeding bother (EPIC item 15e). To evaluate the correlation between rectal bleeding based on PROMs versus CTCAE assessment, Spearman’s rank correlation was applied. *p*-values < 0.05 were considered statistically significant.

## Results

### Overview

**Figure 2:**
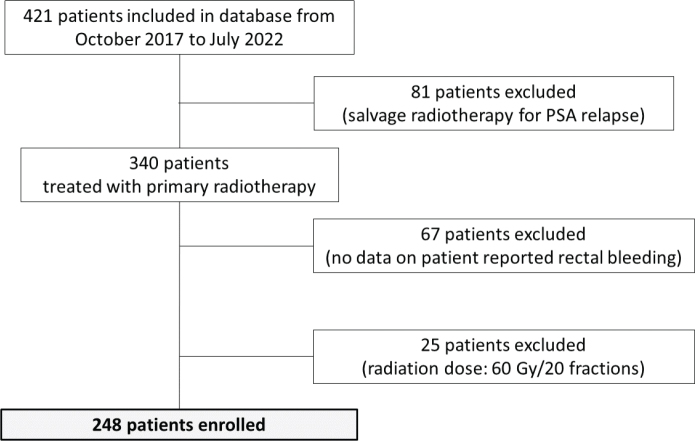
Flowchart of patient enrollment.

A total of 248 patients (median age: 70 years [IQR: 66–73]) with stage T1-T3N0M0 were investigated (Figure 2). Patient, disease, and dosimetric characteristics are listed in Table 1. Median follow-up was 18 months (range 1–61 months). Eleven patients had disease recurrence, and eight patients died during follow-up. Rectum dose constraint of *V*_74 Gy_ ≤ 12% was reached in all patients except one with a *V*_74 Gy_ of 12.6%.

**Table 1:** Patient disease and dosimetric characteristics.

Patient characteristics at baseline		*N* = 248	
Age, median (IQR), years		70	(66–73)
WHO performance status, *n* (%)	*0*	208	(85)
	*1*	34	(14)
	*2*	3	(1)
	*Unknown*	4	
Charlson Comorbidity Index, *n* (%)	None	0	
	Mild (score 1–2)	64	(27)
	Moderate (score 3–4)	142	(60)
	Severe (score ≥ 5)	31	(13)
	*Unknown*	11	
Body mass index, *n* (%)	Underweight (BMI < 18.5)	1	(<1)
	Normal (BMI 18.5–24.9)	56	(23)
	Overweight (BMI 25.0–29.9)	111	(46)
	Obese (BMI 30.0–34.9)	52	(22)
	Extremely obese (BMI ≥ 35.0)	20	(8)
	*Unknown*	8	
Diabetes, *n* (%)	No	201	(86)
	Yes (three with end-organ damage)	33	(14)
	*Unknown*	14	
Hemorrhoids, *n* (%)	No	202	(82)
	Yes (CTCAE I or II)	42	(18)
	*Unknown*	4	
Obstipation, *n* (%)	No	221	(90)
	Yes (CTCAE I or II)	25	(10)
	*Unknown*	2	
Tobacco, *n* (%)	Non-smoker	73	(31)
	Previous smoker	127	(54)
	Smoker	37	(16)
	*Unknown*	11	
Anticoagulants or antiaggregants, *n* (%)	No	130	(53)
	Yes	116	(47)
	*Unknown*	2	
	*Anticoagulants*	45	(39)
	*Antiaggregants*	71	(61)
**Disease characteristics**			
Prostate-specific antigen, median (IQR), μg/l		15	(8.9–32)
Gleason score, median (IQR)		7	(7–8)
	*Unknown*	5	
T-stage, *n* (%)	Tx	1	(<1)
	T1a-T1c	27	(11)
	T2a-T2c	40	(16)
	T3a	110	(44)
	T3b	70	(28)
Androgen deprivation therapy, *n* (%)	None	12	(5)
	6 months	24	(10)
	36 months	212	(85)
**Dosimetric characteristics**			
Prostate volume, median (IQR), cm^3^		42	(34–55)
	*Unknown*	27	
Radiotherapy targets, *n* (%)	Prostate boost (≤T3a)	172	(69)
	Prostate and seminal vesicles boost (T3b)	76	(31)
Dose-volume metrics, median (IQR), Gy			
≤T3a (prostate boost)			
Prostate	*D*_98%_	74.4	(74.2–74.5)
Prostate	*D*_95%_	75.3	(75.1–75.5)
Prostate	*D*_50%_	78.6	(78.3–78.8)
Prostate	*D*_2%_	81.5	(81.1–81.8)
Seminal vesicles	*D*_98%_	54.6	(53.7–54.6)
Seminal vesicles	*D*_95%_	54.8	(54.4–55.5)
Seminal vesicles	*D*_50%_	64.9	(60.0–71.6)
Seminal vesicles	*D*_2%_	80.8	(80.3–81.2)
T3b (prostate and seminal vesicles boost)			
Prostate	*D*_98%_	74.4	(74.3–74.6)
Prostate	*D*_95%_	75.3	(75.2–75.6)
Prostate	*D*_50%_	78.6	(78.2–78.9)
Prostate	*D*_2%_	81.6	(81.1–82.0)
Seminal vesicles	*D*_98%_	74.2	(73.6–74.5)
Seminal vesicles	*D*_95%_	75.2	(74.6–75.4)
Seminal vesicles	*D*_50%_	78.6	(78.1–79.0)
Seminal vesicles	*D*_2%_	81.6	(81.2–82.0)
Rectum volume, median (IQR), cm^3^		57	(45–68)
Rectum *V*_70Gy_, median (IQR), cm^3^		6.5	(4.6–8.7)
Rectum *V*_70Gy_, (%) median (IQR)		12	(9–15)
Rectum *V*_74Gy_, median (IQR), cm^3^		4.4	(3.3–5.9)
Rectum *V*_74Gy_, (%) median (IQR)		8	(6–11)
Number of patients with rectum *V*_74Gy_ > 12%		1	

BMI: body mass index; CTCAE: common terminology criteria for adverse events; IQR: interquartile range; *V*_70Gy_: volume receiving ≥70 Gy; *V*_74Gy_: volume receiving ≥74 Gy.

Among patients with no PROM data (*N* = 67), 64 patients were assessed with CTCAE. Seven patients had grade 1 rectal bleeding mainly reported at EOT.

### Prevalence of rectal bleeding

**Figure 3:**
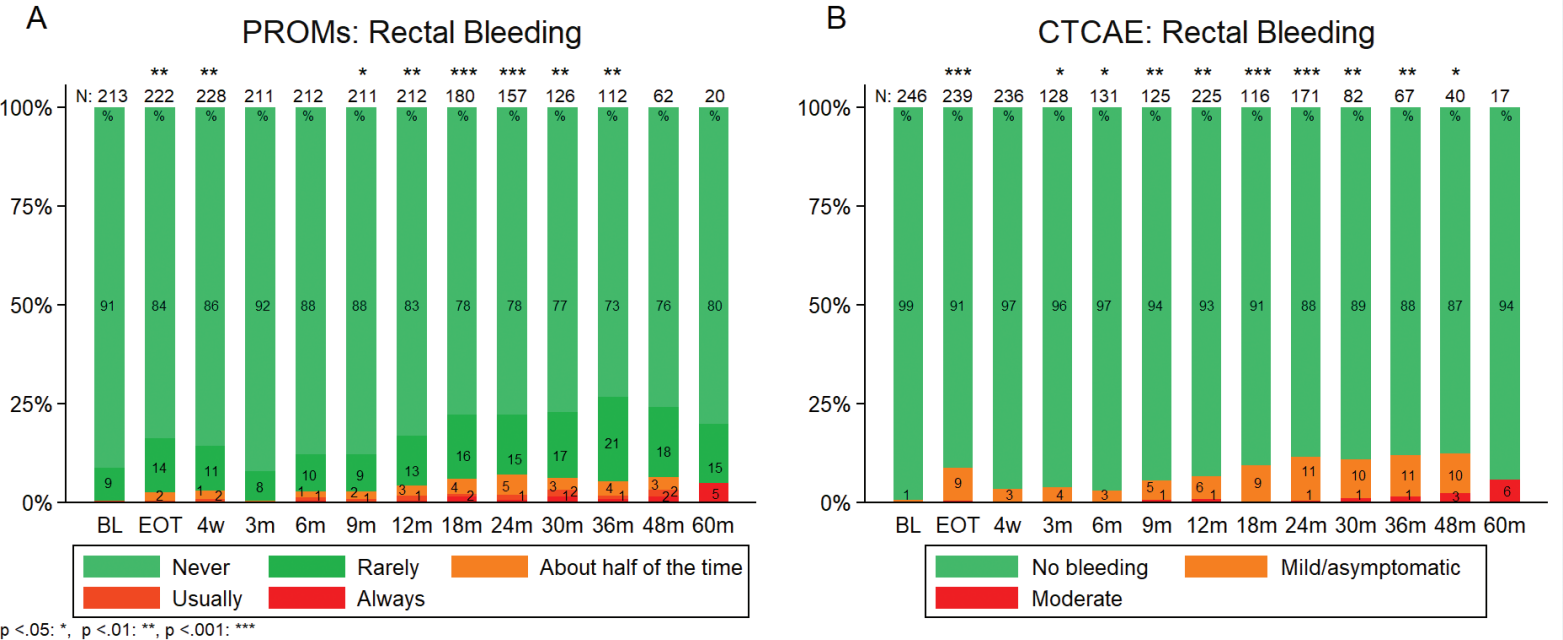
Prevalence (%) for rectal bleeding at baseline and follow-up. p-values based on Wilcoxon signed-rank test between baseline and follow-up. A: Patient-reported outcome (EPIC), B: Staff-assessed morbidity (CTCAE). Numbers inside each bar indicate percentage of rectal bleeding. BL: baseline, EOT: end of treatment, m: months, N: number of patients at different time point, RB: rectal bleeding, w: weeks.

Figure 3 depicts the prevalence of rectal bleeding at every time point for both PROM and CTCAE registrations. There was a statistically significant difference in prevalence of patient-reported rectal bleeding from BL to EOT (*p* = 0.005) as well as from BL to 4 weeks (*p* = 0.008) and 9 (*p* = 0.032), 12 (*p* < 0.001), 18 (*p* < 0.001), 24 (*p* = 0.003), 30 (*p* = 0.006), and 36 (*p* = 0.001) months of follow-up (Figure 3A). Similar results were obtained when staff-reported rectal bleeding was analyzed. During follow-up, significantly higher prevalence was found compared to BL levels except from 4 weeks and 60-month follow-up (Figure 3B). The frequencies of rectal bleeding throughout the follow-up period for each individual patient are presented in Figure S1.

### Cumulative hazard ratio for rectal bleeding and Cox regression analysis

**Figure 4:**
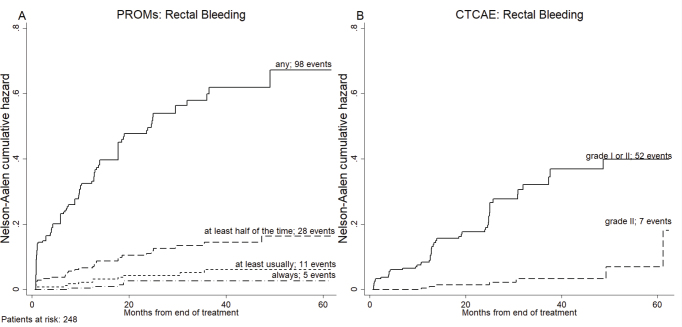
Cumulative hazard function curves for patient-reported rectal bleeding (A) and staff-assessed rectal bleeding (B), ordered by frequency/severity of rectal bleeding at end of treatment and up to 60 months of follow-up.

The Nelson-Aalen cumulative hazard curves in Figure 4A illustrate the risk of having at least one PROM rectal bleeding from EOT and throughout the rest of the follow-up period ordered by the severity/frequency of bleeding from ‘any bleeding’, ‘at least half of the time’, ‘at least usually’ to ‘always’. In Figure 4B, the cumulative hazard ratio (HR) for CTCAE-reported rectal bleeding, either as grade 1 and 2 or as grade 2 alone (no CTCAE grade > 2 rectal bleeding was observed), is presented.

The results from the univariate and multivariate Cox regression analyses are demonstrated in Table 2. In the multivariate analysis, larger prostate volume was associated with higher risk of rectal bleeding (HR: 1.01 [confidence interval, CI: 1.0; 1.03], *p* = 0.02). The likelihood of rectal bleeding was lower in patients with BMI 25–29.9 (44 out of 111 patients reported rectal bleeding, HR: 0.54 [CI: 0.30; 0.98], *p* = 0.044) and BMI > 29.9 (24 out of 72 patients had rectal bleeding, HR: 0.40 [CI: 0.20; 0.79], *p* = 0.008) compared to patients with BMI < 25 (28 out of 57 patients had rectal bleeding). CTCAE hemorrhoids and tobacco (smoker vs. no smoker) (HR: 0.52 (CI: 0.26; 1.0), *p* = 0.06) also met the criteria (*p* < 0.1) for multivariate testing but did not reach statistical significance in the test (Table 2).

**Table 2:** Univariate and multivariate Cox regression analyses of prognostic factors for patient-reported rectal bleeding after prostate cancer radiotherapy.

COX regression, *N* = 248	Univariate			Multivariate		
	Hazard ratio	95% CI	*P*	Hazard ratio	95% CI	*p*
**Patient characteristics at baseline**						
Age	0.98	0.95; 1.0	0.58			
WHO performance status						
0	Ref.					
1	0.88	0.46; 1.7	0.7			
2	1.93	0.59; 6.3	0.27			
Charlson Comorbidity Index						
Mild (1–2)	Ref.					
Moderate (3–4)	1.2	0.74; 2.0	0.43			
Severe (≥5)	1.5	0.73; 2.9	0.29			
Body mass index						
Normal (BMI < 24.9)	Ref.					
Overweight (BMI 25.0–29.9)	0.75	0.47; 1.2	0.23	0.54	0.30; 0.98	**0.044**
Obese (BMI > 29.9)	0.61	0.37; 1.0	0.08	0.40	0.20; 0.79	**0.008**
Diabetes						
No	Ref.					
Yes	0.84	0.43; 1.6	0.62			
Tobacco						
Non-smoker	Ref.			Ref.		
Previous	0.77	0.50; 1.2	0.23	0.96	0.55; 1.7	0.89
Smoker	0.53	0.25; 1.1	0.08	0.64	0.25; 1.3	0.18
Anticoagulant or antiaggregants						
No	Ref.					
Yes	1.1	0.73; 1.6	0.68			
**Disease characteristics at baseline**						
Prostate specific antigen	0.99	0.98; 1.0	0.27			
Gleason score	1.0	0.82; 1.3	0.81			
T-stage						
T1a-T1c	Ref.					
T2a-Tc	1.13	0.53; 2.7	0.76			
T3a	1.1	0.60; 2.1	0.73			
T3b	0.82	0.40; 1.7	0.58			
Androgen deprivation therapy						
None or 6 months	Ref.					
36 months	1.2	0.68; 2.0	0.59			
**Dosimetric characteristics**						
Prostate volume	1.00	1.0; 1.02	0.08	1.01	1.0; 1.03	**0.02**
Radiotherapy targets						
Prostate alone	Ref.					
Prostate + seminal vesicles	0.89	0.57; 1.4	0.61			
Rectum *V*_70Gy_	1.02	0.97; 1.1	0.42			
Rectum *V*_74Gy_	1.03	0.96; 1.1	0.39			
**Staff-assessed outcomes during follow-up**						
Hemorrhoids						
No	Ref.			Ref.		
Yes (CTCAE I-II)	1.7	0.9; 3.0	0.10	1.9	0.95; 3.6	0.07
Obstipation						
No	Ref.					
Yes (CTCAE I-III)	1.37	0.8; 2.5	0.29			

BMI: body mass index; CI: confidence interval; CTCAE: common terminology criteria for adverse events; Ref.: Reference.

Similarly, when the absolute rectal volume in terms of *V*_70 Gy_ and *V*_74 Gy_ was analyzed, no associations with rectal bleeding were found. In the univariate analysis, HRs were 1.0 (95% CI: 0.98; 1.1) *p* = 0.14 for *V*_70 Gy_ and HR 1.1 (0.98; 1.2) *p* = 0.14 for *V*_74 Gy_, but it did not reach statistical significance in a multivariate analysis (*p* = 0.76 (*V*_70 Gy_) and *p* = 0.95 (*V*_74 Gy_)).

### Patient-reported rectal bleeding and quality of life

At median follow-up, 5% reported bothersome rectal bleeding defined as a moderate (7 patients [4%]) or big problem (2 patients [1%]). To evaluate the clinically significant bother of rectal bleeding, we compared any PROMs on rectal bleeding (EPIC item 11) with quality of life assessment (EPIC item 15e) at the same time point (i.e. patients were presented multiple times). Low prevalence of rectal bleeding was mostly rated as ‘no’ or as a ‘tiny’ problem as opposed to more frequently reported rectal bleeding (total Pearson’s chi^2^ = 232.5, *p* < 0.001) (Table S1).

### Correlation between PROM and CTCAE

There was a moderate agreement between PROMs and CTCAE for rectal bleeding (Spearman’s rho = 0.44, *p* < 0.001).

### Endoscopic results

Fifty-five out of 98 patients (56%) who reported rectal bleeding were investigated with endoscopy. In 29 (53%) patients, verified signs of radiation proctitis were found, whereas other etiologies for rectal bleeding (e.g. diverticulosis, hemorrhoids, and polyps) were present in the rest of the patients (Figure S2).

## Discussion

This prospective study shows that rectal bleeding is a common adverse event following EBRT for prostate cancer. Furthermore, rectal bleeding may be bothersome with an impact on quality of life. However, in our study, other factors than radiotherapy dose-volume parameters were associated with rectal bleeding, and moreover, among those with rectal bleeding, who underwent endoscopic examination, only half of the patients were diagnosed with treatment-related radiation proctitis, while other causes not related to radiotherapy were diagnosed in the remaining patients.

Analysis of risk factors for rectal bleeding showed that patients with overweight (BMI: 25–29.9) or obesity (BMI > 29.9) had lower risk of rectal bleeding compared to patients with normal BMI (BMI < 25). Importantly, no associations between rectal bleeding and anticoagulating/antiaggregant therapy, irradiated rectal volume, diabetes, or ADT were found.

In our study and in the study by Fiorino et al., median follow-up times were of 18 months and 24 months, respectively. This is contrary to the study by Tucker et al., who reported late rectal toxicity at 5 years after a median follow-up of 7.2 years [12, 16]. The short follow-up time in our study and the study by Fiorino et al. is a limitation when late toxicity after radiotherapy is investigated. However, in a large study by Mazeron et al., the incidence of rectal bleeding at 9 months was comparable to the results that were observed after 5 years of follow-up among 960 patients who underwent radiotherapy for cervical cancer [22]. These findings suggest that radiotherapy-related late rectal bleeding tends to manifest early, and that a substantial proportion of all the patients with rectal bleeding are identified even within a shorter follow-up period.

Previous studies have shown that especially high-dose regions within the rectum is a risk factors for rectal bleeding after prostate cancer radiotherapy, but in these studies, it has also been shown that patients, in general, have a background risk for rectal bleeding due to other factors that are independent of rectal dose [14, 23]. This finding is in agreement with our data where rectal bleeding was prevalent during follow-up, and no impact of radiation dose and irradiated rectal volume was found.

Generally, bothersome rectal bleeding is reported by 1%–2% of the patients after prostate cancer radiotherapy [2, 24, 25]. This number is slightly lower compared to the results from our study and may be attributed to different follow-up times or patient population [2, 24]. Moreover, differences in radiation doses and techniques, study design, and methodology have to be taken into consideration [26]. Finally, the majority of studies reporting PROM on rectal bleeding uses EPIC-26, which only encompasses quality of life measures and not frequency measures, which makes direct comparison with the data from the present study difficult [9, 24, 25].

A moderate correlation between PROM- and CTCAE-assessed rectal bleeding was found in the present study. This is in agreement with previous studies reporting both patient-reported and staff-assessed morbidity [27]. However, in the present study, the prevalence of any rectal bleeding was almost twice as high using PROM as compared to staff-assessed morbidity using CTCAE. This highlights the importance of incorporating patients experience, as well as that direct comparisons between CTCAE and PROM are difficult [28].

The cumulative incidence of rectal bleeding was 2–3 times higher than the prevalence, which underlines that any conclusion regarding risk factors for rectal bleeding using Cox regression analyses should be done with caution. The difference in cumulative incidence and prevalence can be attributed to the fact that morbidity including rectal bleeding has a fluctuating manifestation pattern as a result of a self-limiting nature in some patients or successful morbidity treatments in other patients (Figure S1). Furthermore, rectal bleeding may be due to other causes than radiotherapy. This is also demonstrated in the present study as well as in agreement with previous studies where causes without relation to radiotherapy were diagnosed in half of all the patients who had an endoscopy due to rectal bleeding.

When the impact of prostate volume on rectal bleeding was analyzed, there was an association between increasing volumes and risk of bleeding. Even though a correlation between prostate volume and bleeding seems obvious from a clinical point of view, these results have to be interpreted with caution due to the low HR, which indicates lack of strong correlation between prostate volume and rectal bleeding. The lack of association between prostate volume and rectal bleeding is generally in agreement with previous studies, where only one study using PROMs found a tendency for higher rectal bleeding among patients with large prostate volumes [29].

The lower risk of rectal bleeding among patients with high BMI could, in theory, be explained by reduced irradiated rectal volume as a result of more adipose tissue in the prostate–rectum interface. Other studies have shown confliction results regarding associations between BMI and rectal morbidity. In one study, BMI had a negative impact on bowel function after radiotherapy [30], while two other found no difference between individuals with normal or high BMI [31, 32].

The use of anticoagulant or antithrombotic medication is an obvious risk factor for rectal bleeding, although this was not the case in our study or in a study by Bae et al. [5], who investigated rectal bleeding in patients with prostate cancer using CTCAE. However, these results are contrary to Choe et al. [7], where a relationship between grade 3 CTCAE rectal bleeding and the use of anticoagulants was found. In this study, the majority of the patient populations was treated with Warfarin, which was not the case in our study [7]. Similarly, in a retrospective study, the use of anticoagulants/antiaggregants was associated with grade 2 CTCAE rectal bleeding [33]. The relationship between rectal bleeding and the use of anticoagulation/antiaggregants is therefore not fully elucidated, although it seems that treatment is mostly associated with severe rectal bleeding, even though we did not find this in our study.

Previously, Tucker et al. investigated a dose–effect relationship for rectal toxicity in more than 1,000 patients diagnosed with prostate cancer in the RTOG 94-06 trial from 42 different institutions [14]. The patients were treated with different 3D conformal treatment techniques, which consequently resulted in large variations in rectal dose-volume histograms. The results of the study indicated that intermediate rectal doses did not have any impact on rectal toxicity, while *V*_75 Gy_ was the only dose-volume parameter that was significantly associated with grade ≥ 2 late rectal toxicity. In this study, the authors concluded that patients with a *V*_75 Gy_ less than 12% had a risk for rectal toxicity similar to a background population, while the incidence increased when more than 12% of the rectum was treated beyond a dose of 75 Gy [14]. Here, also 8%–10% of the patients with *V*_75 Gy_ ≤ 12% experienced rectal bleeding, suggesting an a priori risk of rectal bleeding independent of rectal dose.

Based on these findings, we introduced *V*_74 Gy_ ≤ 12% as a rectal dose-constraint in prostate cancer radiotherapy. Subsequently, we were able to deliver the prescribed dose homogeneously to the targets without violation of this constraint in a majority of all patients (Table 1). The fact that most patients had radiotherapy delivered with a low rectal *V*_74 Gy_ may also explain why no rectal dose-response relationship for rectal bleeding was found.

Due to the lack of association between rectal *V*_74 Gy_ and rectal bleeding, we feel reassured that a *V*_74 Gy_ ≤ 12% is safe dose-constraint with regard to rectal bleeding to be used in future patients referred for prostate cancer radiotherapy. Moreover, based on our findings, it may also be concluded that the rectal dose constraints proposed by Fiorino et al. and Dearnaley et al. are too conservative [16, 34], and that a less stringent constraint for *V*_74 Gy_, such as the one used in RTOG 0924 (*V*_74 Gy_ ≤ 15%), can be considered safe in prostate cancer radiotherapy [35].

### Strengths and limitations

Strengths of the study include its prospective design and the relatively large and well-described patient population as well as the use of patient-reported outcomes.

The lack of a control group that did not receive radiotherapy is a limitation. Furthermore, the statistical approach of using cox-regression analysis in evaluating endpoints that usually are self-limited should be taken into consideration. As previously described, cox proportional hazard models exaggerate rates of the endpoint due to the assumption that all events are permanent. This leads to different consequences. First, any development of the event is not described, for example, increased or lower frequency of rectal bleeding is not illustrated. Second, it is unknown whether a specific risk factor is related to higher or more severe rectal bleeding. In this analysis, we only demonstrated factors associated with ‘any’ bleeding. The use of other statistical approaches and sub-group analyses may add valuable information to the possible associations, although the risk of confounding increases, and the statistical power is reduced.

## Conclusion

This prospective study has demonstrated that patient-reported rectal bleeding is common after prostate cancer radiotherapy and can affect quality of life. A higher BMI was found to be a protective factor against rectal bleeding. However, no significant correlation was observed between rectal dose-volume constraints and the occurrence of rectal bleeding. These results suggest that using a rectal high-dose constraint of *V*_74 Gy_ ≤ 12% is an adequate threshold to minimize patient-reported rectal bleeding in prostate cancer radiotherapy.

## Supplementary Material

Risk factors for rectal bleeding in prostate cancer after radiotherapy with a validation of current rectal dose constraints

## Author statement

ES: writing original draft, formal analysis, and visualization, CM: conceptualization and investigation, AZ: investigation, MB: data curation and conceptualization, HN: data curation and conceptualization, TA: data curation and conceptualization, BM: data curation and conceptualization and LF: conceptualization, writing review, statistics review, and editing. All authors have contributed to and approved the final manuscript.
