# Supplementary material for: Risk factors for rectal bleeding in prostate cancer after radiotherapy with a validation of current rectal dose constraints
Source: Acta Oncol. 2025 May 8;64:42551. doi: 10.2340/1651-226X.2025.42551 (PMC12079044; doi:10.2340/1651-226X.2025.42551)
Supplement: Risk factors for rectal bleeding in prostate cancer after radiotherapy with a validation of current rectal dose constraints [file AO-64-42551-s1.pdf]

Supplementary material has been published as submitted. It has not been copyedited, or typeset by Acta Oncologica

Supplementary material

Figure S1: Pattern of rectal bleeding frequency for individual patients. Vertical line indicate median follow up.

BL: baseline, EOT: end of treatment, m: months w: weeks

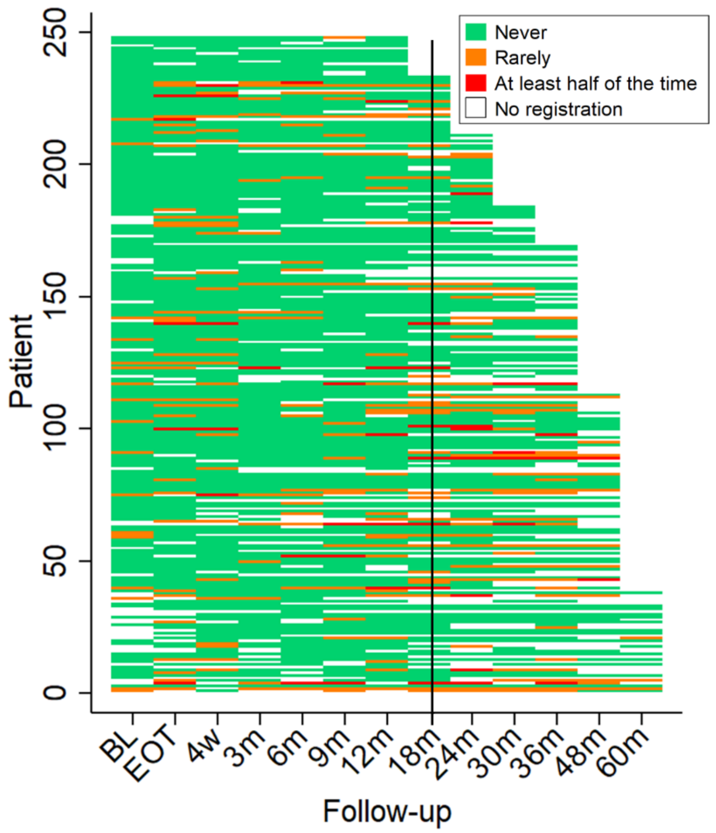

Figure S2: Findings from endoscopy (colonoscopy and sigmoidoscopy) for patients reporting rectal bleeding.

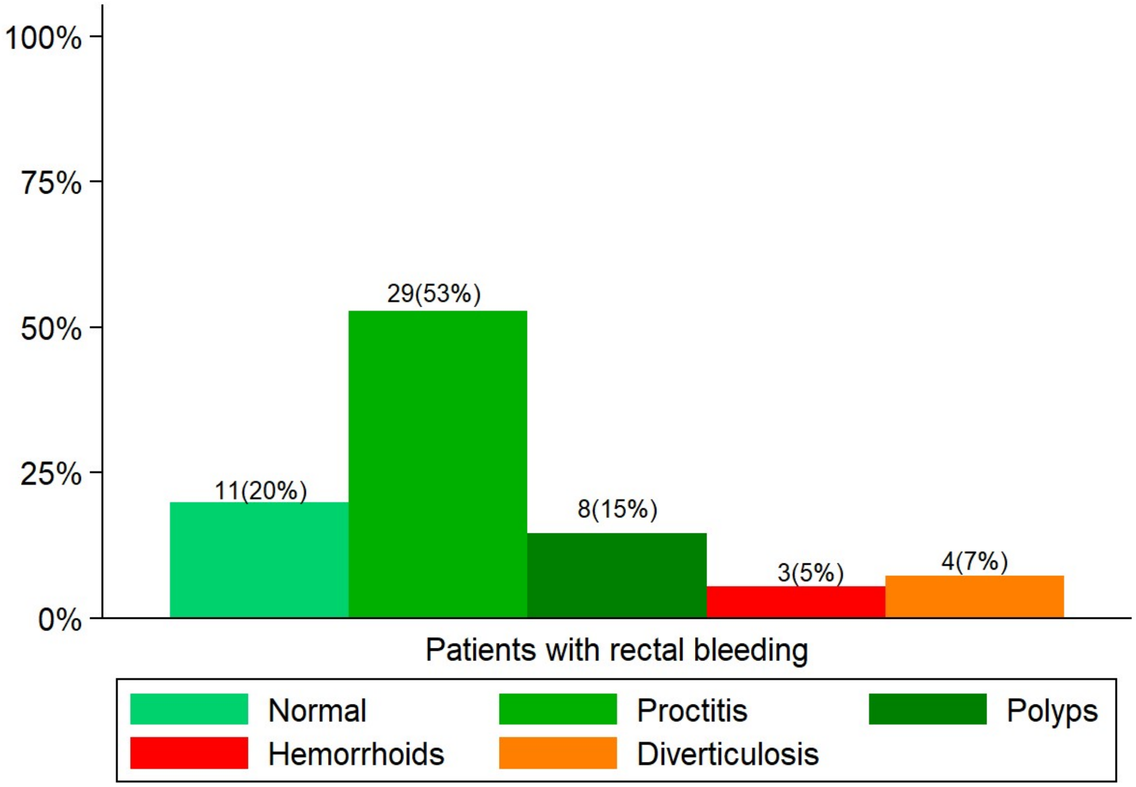

Table S1. All individual registrations of patient reported rectal bleeding with corresponding clinical bother (EPIC item 15e), i.e., patients are present>1 time.

	**Problem**					
	**no problem**	**tiny**	**little**	**moderate**	**huge**	**Total**
**Bleeding, n(%)**						
**Never**	N/A	N/A	N/A	N/A	N/A	N/A
**Rarely**	96 (38)	102 (41)	44(18)	8(3)	0	250
**Half of the time**	2 (4)	9(19)	13(27)	22(46)	2(4)	48
**Usually**	0	0	3(18)	8(47)	6(35)	17
**Always**	4(27)	0	0	2(18)	6(55)	11
						Pearson's chi^2 = 232.5 p<.001
